# Better prioritization to increase research value and decrease waste

**DOI:** 10.1186/s12916-015-0492-3

**Published:** 2015-09-29

**Authors:** Agnes Dechartres, Philippe Ravaud

**Affiliations:** Centre de Recherche Epidémiologie et Statistique, INSERM U1153, Paris, France; Centre d’Epidémiologie Clinique, Hôpital Hôtel-Dieu, Assistance Publique-Hôpitaux de Paris, 1 place du Parvis Notre Dame, 75004 Paris, France; Faculté de Médecine, Université Paris Descartes, Sorbonne Paris Cité, Paris, France; French Cochrane Centre, Paris, France; Department of Epidemiology, Mailman School of Public Health, Columbia University, New York, NY USA

**Keywords:** Randomized controlled trials, Knowledge gaps, Planning, Research agenda, Waste

## Abstract

In a recent study published in *BMC Medicine*, Singh Ospina and colleagues outlined the important gaps between ongoing research and research needs in the field of endocrinology. Many recommendations from clinical practice guidelines are based on a low level of evidence, thereby resulting in research gaps. Despite the publication of around 25,000 randomized controlled trials each year, ongoing research does not cover most of these gaps. In contrast, trials are planned when sufficient data are already available for decision making, which results in redundant research and exposes patients to unnecessary risks. This lack of prioritization contributes to the enormous problem of waste in research. A systematic approach to accumulate the available body of evidence is necessary to determine when we have sufficient evidence and when we have knowledge gaps, defined as research questions with no or a low level of evidence available. Systematic registration of research gaps and their prioritization may help to organize future research. Some initiatives exist, but they need to be generalized.

Please see related research: http://www.biomedcentral.com/1741-7015/13/187

## Background

### Waste related to poor planning and prioritization of research

In 2009, Chalmers and Glasziou highlighted the enormous problem of waste in research, estimating that up to 85 % of research investment is wasted [1]. Waste occurs at all stages of research [2–7] and particularly affects planning and prioritization. We have increasing evidence that many trials address low-priority questions that are poorly related to the burden of disease [8] and patient or physician needs [9, 10], do not address patient-important outcomes [11] or use an inadequate comparator. For example, in rheumatology, few trials compare biologically active drugs against each other; comparisons against placebo represent 80 % of trials registered at ClinicalTrials.gov [12]. This lack of head-to-head trials does not allow for answering the pragmatic question raised by patients and their physicians: for this particular disease, which treatment is most effective? Also, it exposes patients to unnecessary risks [12, 13]. Last but not least, trials are frequently planned regardless of the existing evidence. More than 50 % of trial protocols do not refer to systematic reviews [14]. Many trials are planned when sufficient data are already available for decision making, which results in redundant research and exposes patients to unnecessary risks [3]. In contrast, trials are not planned when they are needed to fill research gaps, as highlighted by Singh Ospina and colleagues in a recent study published in *BMC Medicine* [15]. The authors defined research gaps as clinical questions with a very low level of evidence according to the Grading of Recommendations Assessment, Development and Evaluation (GRADE) approach from clinical practice guidelines [15].

### The worrying proportion of clinical practice guidelines based on a poor level of evidence

In many clinical practice guidelines, few of the recommendations are based on high-level evidence [16–18]. For example, a 2009 study published in *JAMA* showed that 11 % of the recommendations in clinical practice guidelines from the American College of Cardiology (ACC) and the American Heart Association (AHA) were considered to be based on a high level of evidence [18]. Identifying clinical questions for which only low-level evidence is available should, in theory, help in planning future clinical trials focusing on these areas. Nevertheless, a study evaluating practice guidelines from the Infectious Diseases Society of America showed no improvement in proportion of recommendations with a high level of evidence over time [17]. It is very worrying that, despite the publication of around 25,000 randomized controlled trials each year, clinical practice guidelines continue to rely mostly on a poor level of evidence, with potentially serious consequences for patient care [19]. Singh Ospina and colleagues used ClinicalTrials.gov to assess the response in terms of new, active studies conducted for research questions with a very low quality of evidence according to the Endocrine Society clinical practice guidelines [15]. The authors found active studies for only one of five recommendations, which suggests that ongoing research does not sufficiently adapt to fill knowledge gaps in endocrinology.

### Identifying gaps to decrease waste

The research community is becoming increasingly concerned by these issues. We need to add incremental value to existing evidence by a better connection to future research. A first step is to systematically identify research gaps. As outlined by Singh Ospina and colleagues, clinical practice guidelines could be helpful. In the same way, systematic reviews, by synthesizing the available body of evidence, have a key role to play. The Cochrane Collaboration clearly recommends that review authors systematically comment on the need for further research in a separate section of the review, called “Implications for research” [20]. Then, a second step would be to record research gaps. Some initiatives already exist. The UK Database of Uncertainties about the Effects of Treatments (UK DUETs), established by the National Institute for Health and Care Excellence (NICE), publishes treatment uncertainties reported by patients and clinicians and derived from research recommendations and systematic reviews [21]. The Agency for Health Research and Quality (AHRQ) has developed an approach to identify and prioritize future research needs to be used by researchers and funders to help improve the body of comparative effectiveness evidence that would be useful for decision makers [22]. However, there is not enough information about these burgeoning initiatives. Nothing is done to facilitate the registration of research gaps from different resources and there is no particular incentive for review authors and those of clinical practice guidelines to do so. A joint initiative to centralize registration of gaps in a simple and comprehensible way would be very helpful to enhance communication between researchers, physicians and funders. Finally, the response in terms of new active studies conducted should be monitored to assess the adequacy between ongoing research and knowledge gaps. With the requirement to register trials at ClinicalTrials.gov or in other registries, assessing the clinical trial enterprise and monitoring whether ongoing research fits with research needs has become easier.

## Conclusions

Better prioritization of future research is necessary to increase research value in a context of limited human and monetary resources. Some initiatives exist to register and prioritize research gaps. Such efforts should be encouraged and generalized to realign future studies with the existing body of evidence.

## Abbreviations

ACC: American College of Cardiology
AHA: American Heart Association
AHRQ: Agency for Health Research and Quality
NICE: National Institute for Health and Care Excellence
UK DUETs: UK Database of Uncertainties about the Effects of Treatments
